# Diagnosis implications of the whole genome sequencing in a large Lebanese family with hyaline fibromatosis syndrome

**DOI:** 10.1186/s12863-017-0471-0

**Published:** 2017-01-19

**Authors:** Zahraa Haidar, Ramzi Temanni, Eliane Chouery, Puthen Jitesh, Wei Liu, Rashid Al-Ali, Ena Wang, Francesco M Marincola, Nadine Jalkh, Soha Haddad, Wassim Haidar, Lotfi Chouchane, André Mégarbané

**Affiliations:** 1 Unité de Génétique Médicale, Faculté de Médecine, Université Saint-Joseph, Beirut, Lebanon; 2 Bioinformatics Division, Sidra Medical & Research Center, Doha, Qatar; 3 Genomics Core Laboratory, Translational Medicine Division, Sidra Medical & Research Center, Doha, Qatar; 4 Research office, Sidra Medical & Research Center, Doha, Qatar; 5 Department of Radiology, Hotel Dieu de France University hospital–Beirut, Beirut, Lebanon; 6 Department of General surgery, Dar Al Amal University Hospital-Baalbeck, Baalbeck, Lebanon; 7 Laboratory of Genetic Medicine and Immunology, Weill Cornell Medicine-Qatar, Doha, Qatar; 8 Institut Jérôme Lejeune, 37, rue des Volontaires, Paris, 75015 France

**Keywords:** Juvenile hyaline fibromatosis, Infantile systemic hyalinosis, Hyaline fibromatosis syndrome, Whole genome sequencing, Anthrax toxin receptor 2 gene

## Abstract

**Background:**

Hyaline fibromatosis syndrome (HFS) is a recently introduced alternative term for two disorders that were previously known as juvenile hyaline fibromatosis (JHF) and infantile systemic hyalinosis (ISH). These two variants are secondary to mutations in the anthrax toxin receptor 2 gene (*ANTXR2)* located on chromosome 4q21. The main clinical features of both entities include papular and/or nodular skin lesions, gingival hyperplasia, joint contractures and osteolytic bone lesions that appear in the first few years of life, and the syndrome typically progresses with the appearance of new lesions.

**Methods:**

We describe five Lebanese patients from one family, aged between 28 and 58 years, and presenting with nodular and papular skin lesions, gingival hyperplasia, joint contractures and bone lesions. Because of the particular clinical features and the absence of a clinical diagnosis, Whole Genome Sequencing (WGS) was carried out on DNA samples from the proband and his parents.

**Results:**

A mutation in *ANTXR2* (p. Gly116Val) that yielded a diagnosis of HFS was noted.

**Conclusions:**

The main goal of this paper is to add to the knowledge related to the clinical and radiographic aspects of HFS in adulthood and to show the importance of Next-Generation Sequencing (NGS) techniques in resolving such puzzling cases.

## Background

Juvenile hyaline fibromatosis (JHF, OMIM # 228600) is a rare inherited autosomal recessive disorder [1] that was first described by McMurray as *Molluscom fibrosum* [2]. Clinically, it is characterized by skin lesions (nodules and/or pearly papules); gingival hyperplasia; joint contracture; abnormal growth of hyalinized fibrous tissues of the head, neck and extremities; and bone lesions [3]. Affected individuals are usually asymptomatic at birth, the onset of clinical signs occurs between 3 months and 4 years of age [4, 5], and these signs increase in severity with age [6, 7]. Most people with JHF survive until the fourth decade of life [8].

Infantile systemic hyalinosis (ISH, OMIM # 236490), another rarer disorder, shares many similarities with JHF [9, 10]. It is characterized by a more severe presentation than JHF and has an early onset (first weeks or months of life) and symptoms that include failure to thrive, short stature, diffuse thickening of the skin, hyperpigmented plaques over the joints, visceral involvement, persistent diarrhea and recurrent infections, and death usually occurs within the first 2 years of life [11–13].

Deleterious mutations of Anthrax toxin receptor 2 gene, (*ANTXR2*; OMIM # 608041) have been shown to cause both JHF and ISH [14–16]. The presence of a significant overlap at the molecular, histological and clinical levels between JHF and ISH have led to the adoption by Nofal et al. of an unifying taxonomy of “hyaline fibromatosis syndrome or HFS”, signifying that both entities represent the same disorder but with different degrees of severity [10, 17]. *ANTXR2* encodes a 55 kDa type I transmembrane (TM) protein which comprises an extracellular N-terminal von Willebrand A (vWA) domain, followed by an immunoglobulin-like domain (Ig-like), a TM domain and a cytosolic tail [18, 19]. This protein is responsible for binding laminin and collagen IV via the vWA domain and the consequent plays a role in basement membrane matrix assembly and endothelial cell morphogenesis [15]. The Ig-like domain contains two disulfide bonds that are essential for proper ANTXR2 localization in the endoplasmic reticulum [18]. The cytosolic tail contains multiple sites for posttranslational modifications such as palmitoylation [20], phosphorylation and ubiquitination [21].

Genotype-phenotype correlation studies have suggested that the mutational spectrum might explain the wide phenotypic variability. Milder phenotypes are associated with in-frame and missense mutations within the cytoplasmic domain, whereas the more severe forms are caused by missense and truncating mutations in the vWA domain and at least one insertion/deletion mutation causing a translational frameshift. However, this correlation is not always constant, thus indicating that modifier genes and/or environmental elements can be involved [15, 22].

Approximately 150 cases of HFS have been reported in the literature [23]. Most of them were diagnosed in early childhood [24], but only a few cases were investigated in adults; the oldest patient was 51 years old [25].

In this paper, we report a large Lebanese family with five HFS patients aged between 28 and 58 years. The oldest patient (58 years) is described here. The aim of this report is to augment the findings related to the clinical, radiographic and differential diagnosis of HFS.

## Methods

### Clinical report

**Fig. 1:**
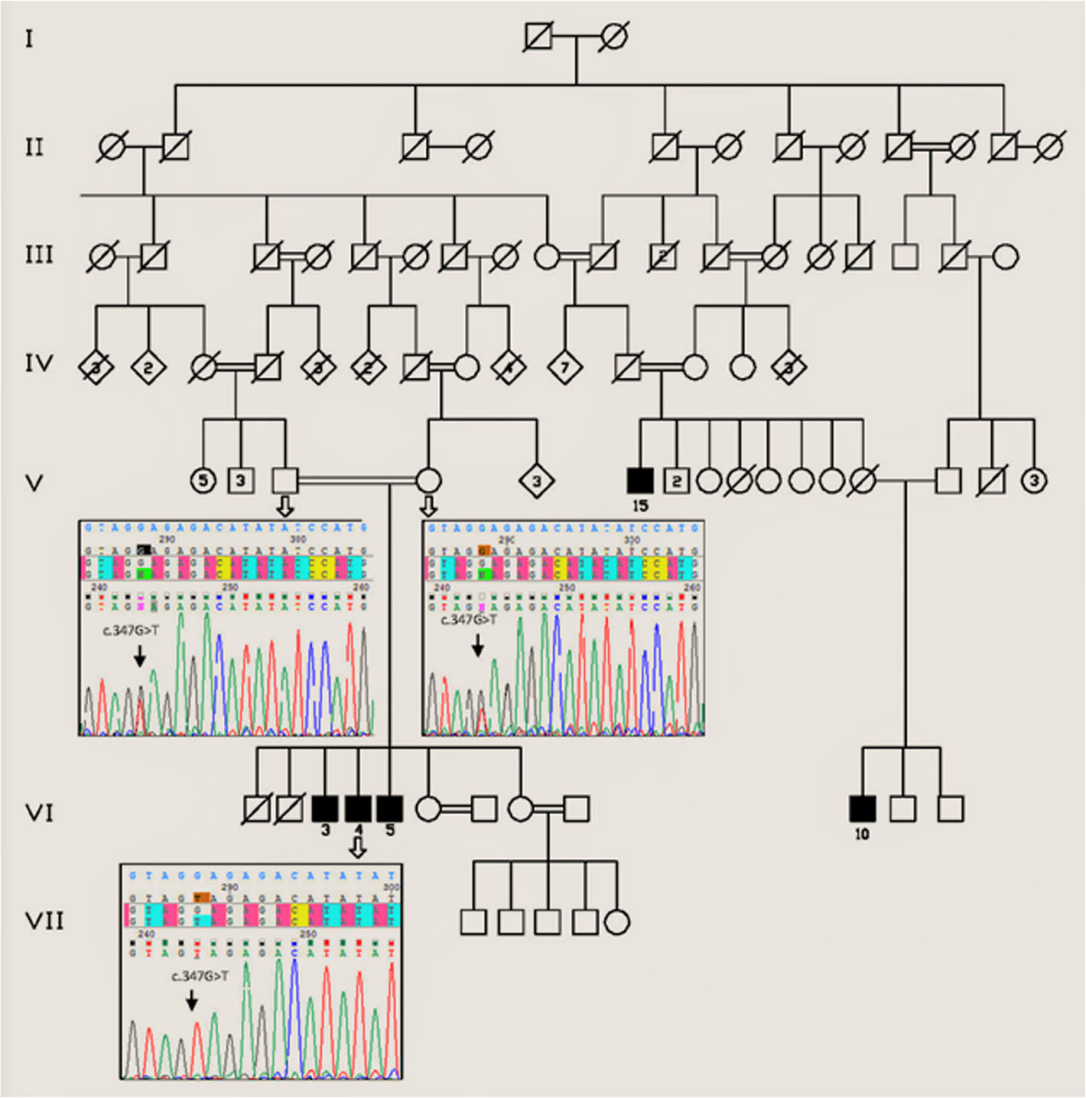
Pedigree chart of the family and genomic DNA sequencing of the proband and both parents. The c.347G > T mutation in *ANTXR2* was homozygous in the proband and heterozygous in the parents

We identified one Lebanese Shiite family with three branches from a small village in North Lebanon (Fig. 1). All five patients were born to healthy consanguineous couples. The pregnancies were not followed medically but were reported to be without complications. For all patients, the skin eruptions and gingival enlargement were first noticed at the age of 6 months, and the nodules continued to gradually increase in number and size.

At the time of physical examination, patient VI-3 was 42 years old, patient VI-4 was 36 years old, patient VI-5 was 30 years old, patient VI-10 was 28 years old and patient V-15 was 58 years old. Patients VI-3, VI-4, VI-10 and V-15 presented with an important postural deformity, and had been in wheelchairs since they were 10 years old, whereas patient VI-5 had a severe retardation of physical growth and development that caused movement difficulty.

The patients were thin with underdeveloped musculatures. Cognitive development, hearing and eyesight were noted to be normal in all patients.

**Fig. 2:**
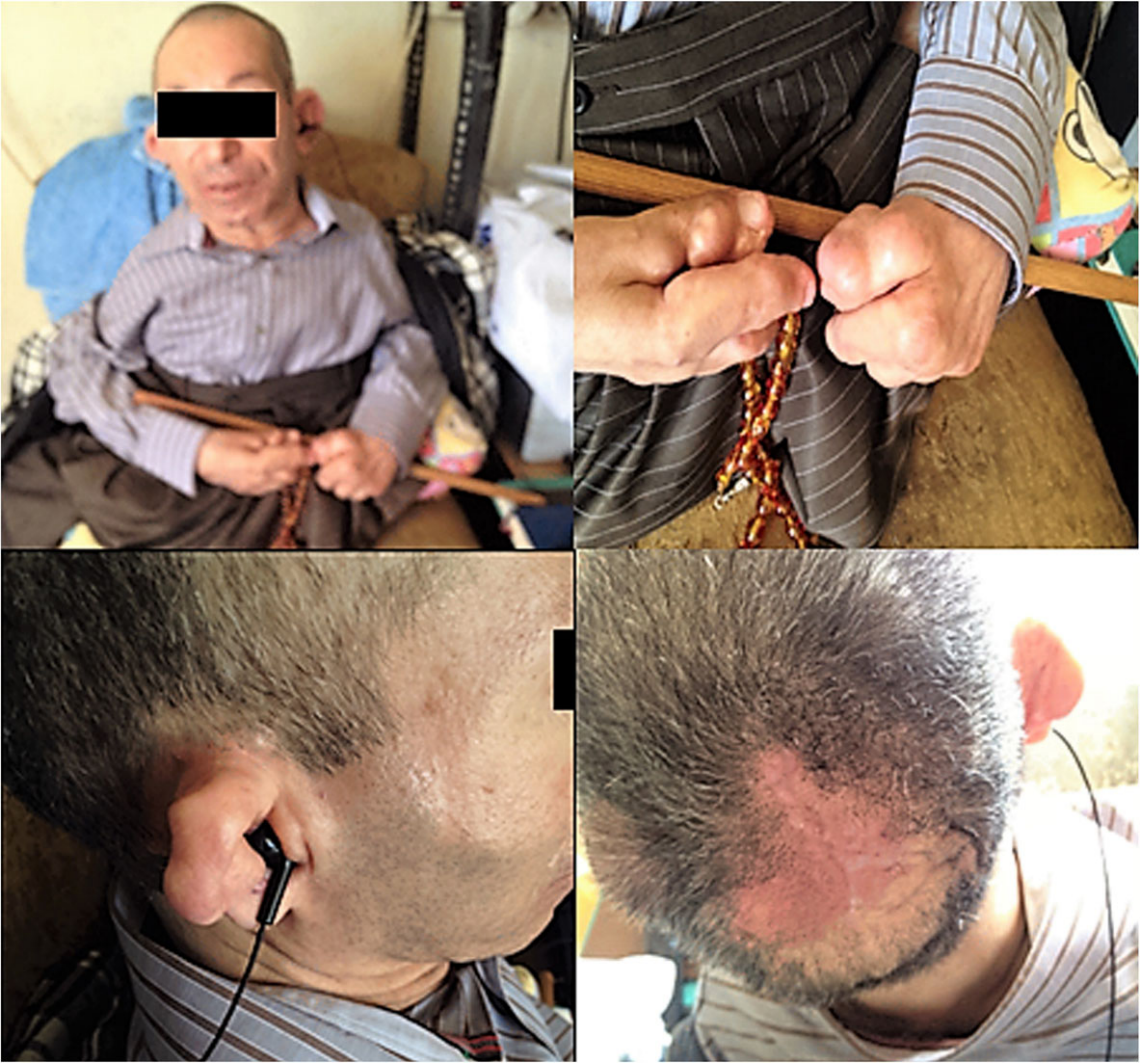
Clinical photographs of the patient V-15. Note the multiple skin nodules distributed on various body regions (mainly, ear and fingers) and flexion contractures of the joints (wrists, knees, ankles and fingers)

Each patient was found to have recurrent, painless, variable-sized nodules over the scalp, ear, lobules, postauricular folds, forehead, nose, upper lip, shoulder, elbows, thorax, chest, back, fingers, perianal area, knee and feet. Small, pearly papules were limited to the chin and paranasal folds. The nose and ears were deformed and bulbous, secondary to numerous tumors. All patients had severe gingival hypertrophy covering the teeth completely. Patients VI-10 and V-15 had flexion contractures of the elbows, and fingers and hips and knees, which resulted in a frog leg position (Fig. 2). Swellings and deformities in the feet, especially in the terminal phalanges of the toes, were also noted. The toenails were thickened.

Hematological and biochemical investigations were within normal limits. Only, patient VI-3 reported having persistent diarrhea since the age of 2 years. The clinical features of the present cases and of ISH and JHF, on the basis of the work of Urbina et al.[10] are shown in Table 1.

**Table 1:** Clinical Features of the patients on the basis of the work of Urbina et al.[10]

	Patient VI-3	Patient VI-4	Patient VI-5	Patient V-15	Patient VI-10	ISH	JHF
Papular skin lesions	+	+	+	+	+	+	+
Thickened skin	-	-	-	-	-	+	-
Gingival hyperplasia	+	+	+	+	+	+	+
Perianal nodules	+	+	+	+	+	+	+
large nodules/tumors	+	+	+	+	+	-	+
Hyperpigmented plaques Joints and bones	-	-	-	-	-	+	-
joint contractures	+	+	+	+	+	+	+
Osteoporosis/osteopenia	+	+	+	+	+	+	+
Osteolysis	+	+	+	+	+	+	+
Persistent diarrhea	+	-	-		-	+	-
Recurrent infections	-	-	-	-	-	+	-
Visceral involvement	-	-	-	-	-	+	-
Short stature	-	-	-	-	-	+	-
Prolonged survival	+	+	+	+	+	-	+

**Fig. 3:**
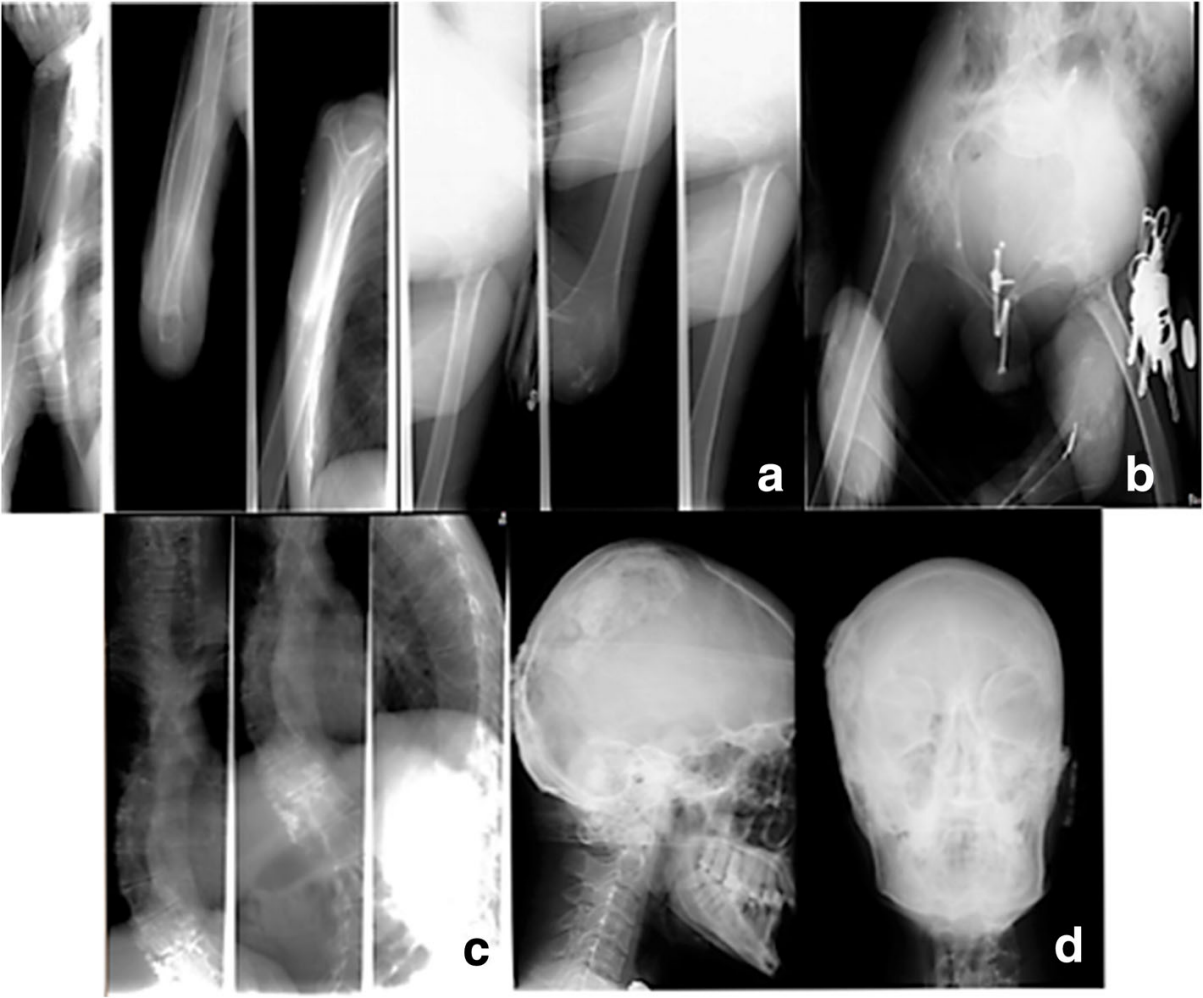
X-rays of patient VI-10 showing (**a**) radial bone bowing and thin diaphyses, (**b**) deformity of the iliac bones, thinned pubic rami, severe narrowing of the hip joints, acetabular protrusion, erosion of joint spaces, coxo-femoral ankylosis, thinned fibula, amyotrophy and cutaneous calcifications, (**c**) thoraco-lumbar scoliosis with paravertebral calcifications at T10, T11 and T12 levels and (**d**) subcutaneous soft tissue calcifications in the pinna of both ears and in the parietal region of the scalp

A skeletal X-ray of patient VI-10 showed subcutaneous soft tissue calcifications in the pinna of both ears and in the parietal region of the scalp, radial bone bowing, thoraco-lumbar scoliosis with paravertebral calcifications at T10, T11 and T12 levels, deformity of the iliac bones, thinned pubic rami, severe narrowing of the hip joints, acetabular protrusion, erosion of joint spaces, coxo-femoral ankylosis, thinned fibula, amyotrophy and cutaneous calcifications (Fig. 3).

The patients refused biopsies of their lesions.

### DNA extraction and Whole Genome Sequencing (WGS)

Genomic DNA was extracted from peripheral blood samples by standard salt-precipitation methods [26]. Whole genome sequencing was carried out on the DNA of patient VI-4 and his parents with a HiSeq 2500 sequencer (Illumina, San Diego, CA, USA) at Sidra Medical and Research Center - Qatar. Genomic libraries were generated from 1 μg of genomic DNA using an Illumina TruSeq DNA PCR-Free Sample Preparation Kit. Genomic DNA was sheared using a Covaris system (Woburn, MA, USA). Isolated DNA fragment ends were blunted, A-tailed and ligated with sequencing adaptors with index sequences. Excess adapters and enzymes were removed using AMPure beads (Beckman Coulter Genomics, Danvers, MA, USA). Indexed libraries were size-selected to the 350 bp range using bead-based capture, and the concentration of amplifiable fragments was determined by qPCR, relative to sequencing libraries with a known concentration. Normalized libraries were clustered on a c-BOT machine, and 125 bp paired-end sequencing was performed on the HiSeq 2500 system.

### WGS data analyses

Raw data were mapped to the human genome reference, build 19 (http://www.broadinstitute.org/ftp/pub/seq/references/Homo_sapiens_assembly19.fasta), using BWA aligner [27] version 0.7.7-r441, and variant calling was performed using GATK [28] version 3.3.2. Rare variant analysis was performed using the xbrowse tool (https://xbrowse.broadinstitute.org/). For the trio, the model of inheritance “autosomal recessive” was selected, with the severity of the variant effect set to ‘moderate to high impact’ (Nonsense, essential splice sites, missense frameshift and in frame), call quality as high (genotype quality > 20 and allele balance ratio > 25%) and allele frequency < 1% in 1000 genomes and The Exome Aggregation Consortium (ExAC) v0.3 datasets. The functional consequences of amino acid substitutions were predicted using various tools [29–32].

### Sanger sequencing

Genomic sequence of *ANTXR2* (NM_058172.5) was obtained from UCSC Genomic Browser on Human. Primers used for PCR amplification were designed using Primer3 software (http://frodo.wi.mit.edu) to amplify the region surrounding the mutation detected by WGS in exon 4. PCR reactions were performed using Taq DNA polymerase (Invitrogen Life Technologies, Carlsbad, CA, USA). PCR fragments were run on 1% agarose gel. The fragments were purified using « *SIGMA-ALDRICH* ^*TM*^ *» kit* and then sequenced using the Big Dye_ Terminator v 1.1 Cycle Sequencing Kit (Applied Biosystems, Foster City, CA, USA). Sequence reaction was purified on Sephadex G50 (Amersham Pharmacia Biotech, Foster City, CA) and then loaded into an ABI 3100 system after the addition of Hidi formamide. Electropherograms were analyzed using Sequence Analysis Software version 5.2 (Applied Biosystems) and then aligned with the reference sequences using ChromasPro v1.7.6.1 (Technelysium, Queensland, Australia).

## Results

WGS identified 2 mutations in *ANTXR2* (NM_058172.5: c.347G > T) and in zinc finger protein 618 (*ZNF618)* (NM_133374.2: c.832G > T). The *ANTXR2* mutation results in a substitution of glycine by valine, and the zinc finger protein 618 (*ZNF618)* mutation leads to a premature stop codon. Both had damaging effects, according to the majority of the effect predictors tested (Table 2).

**Table 2:** Variants identified with the WGS analysis while running an *autosomal recessive* model using xbrowse. Damaging effects of these mutations according to three softwares predictors was tested

Gene	Position	Function	Software prediction
*ANTXR2* (NM_058172.5)	Chr4:80977117 G > T	Missense c.347G > T p. Gly116Val	Polyphen score: 0.99 Polyphen prediction: probably damaging Sift score: 0 Sift prediction: damaging Mutation taster prediction: disease causing Mutation taster score: 0.99
*ZNF618* (NM_133374.2)	Chr9:116794951 G > T	Missense c.832G > T p. Glu278*	

Sanger sequencing confirmed the segregation of the c.347G > T mutation in *ANTXR2* with the disease within the family (Fig. 1). The mutation was homozygous in the affected patients, heterozygous in the parents and heterozygous or not found in the unaffected siblings in this family.

## Discussion

Here, we report five adult patients from a consanguineous Lebanese family, who presented with nodular skin lesions, gingival hyperplasia, joint contractures and bone lesions. By WGS, we identified 2 mutations: a mutation in *ZNF618* (c.832G > T) and a mutation in *ANTXR2* (c.347G > T).

*ZNF618*, also known as *KIAA1952* or *NEDD10*, is a protein-coding gene located on chromosome 9q32 and is implicated in transcriptional regulation. Association studies have demonstrated that *ZNF618* may be involved in the occurrence of cleft lip [33], high blood pressure [34], kidney diseases [35] and, in women, in brachial-ankle pulse wave velocity and arterial stiffness [36, 37]. On the basis of these clinical characteristics, we excluded this gene as a candidate gene.

*ANTXR2*, also called the capillary morphogenesis protein gene-2 (*CMG2*) is located on chromosome 4q21 and is implicated in basement membrane matrix and cell morphogenesis [15]. Mutations of this gene have been found to be responsible for HFS. After reviewing the clinical features of our patients, we considered *ANTXR2* to be a candidate gene responsible for the phenotype of the patients studied here.

A clinical diagnosis of HFS was missed because of the advanced age and status of the patients, the stage of the disease, the severity of the clinical manifestations, and incomplete knowledge of the syndrome’s pathogenesis. The patients reported here had undergone multiple surgeries in infancy for the resection of cutaneous nodules, but long-term regression was unlikely, and the tumors continued to increase in size and number. Their parents stopped treating the lesions, and no follow-up was performed for economic reasons. Biopsies were refused by the patients for many reasons, including pain and the absence of treatment. WGS allowed us to diagnose the disease, assess the genotype-phenotype correlations and offer genetic counseling and prenatal diagnosis to the people of the village.

### Classification of HFS

HFS and inherited systemic hyalinosis represent the same disorder, comprising two variants with severe (ISH) and mild (JHF) forms of the disease.

Gilaberte et al. [6] have proposed 2 major and 3 minor diagnostic criteria for JHF. The major criteria are cutaneous lesions (including nodules, tumors and plaques) and gingival enlargement. The minor criteria include joint contractures, osteolytic lesions and/or cortical erosions, and a family history of JHF. In fact, the presence of persistent diarrhea, hyperpigmented plaques, growth retardation and death within the first 2 years of life are more consistent with ISH. The severity and progression of HFS vary among patients, and hence it is difficult to classify a patient into a single class because many cases of JHF are incorrectly identified as ISH, or vice versa and mutations in the same gene underlie both syndromes. Indeed, Bedford et al. [38] have described a severe form of JHF, with persistent or repeated episodes of diarrhea and death occurring in early infancy after several infections yet with no subcutaneous nodules. Hatamochi et al. [39] have reported a 6-year-old girl who was diagnosed with a severe form of JHF and presented with confluent papules and nodules, recurrent respiratory tract infections and chronic diarrhea since birth. Dhingra et al. [40] have reported a 3-year-old girl who presented with recurrent episodes of diarrhea and was diagnosed as having a case of JHF. ISH patients with atypical prolonged survival have also been reported [41]. Kawasaki et al. [42] have reported an elderly woman with JHF who died from aspiration pneumonia. For these reasons, we prefer to classify our patients as having HFS, which includes both disorders, as proposed by Nofal et al. [17].

In contrast, Nofal et al. [17] have classified HFS into three grades according to the severity of organ involvement: G1: mild, G2: moderate and G3: severe. On the basis of this gradation, the mild type presents with only skin involvement and gingival hypertrophy, the moderate type shows additional joint contractures and bone lesions, and the severe type has manifestations resulting from organ involvement, such as persistent diarrhea and recurrent pulmonary infections. Denadai et al. [22] have added a new lethal grade (G4) for patients with organ failure and/or septicemia. In the family studied here, patients VI-4, VI-5, VI-10 and V-15 can be classified as JHF grade 2 and patient VI-3 as ISH grade 3, thus demonstrating the difficulty of clearly differentiating these subclasses.

### Prevalence

HFS is a rare genetic disorder, but it has been documented in families of different ethnic backgrounds on several continents [11]. The life expectancy of patients with HFS syndrome varies from early death in childhood to normal survivorship. The oldest known patient (58 years) is reported here.

### Diagnosis

The diagnosis of this syndrome is based on the clinical features and/or the presence of a molecular diagnosis.

#### Clinical diagnosis

The clinical features associated with HFS syndrome consist of multiple subcutaneous skin nodules/papules, gingival hypertrophy and joint contractures and may be accompanied by systemic symptoms.

The specific pathogenesis of HFS also remains unclear, but some authors have suggested that it results from an abnormality in type IV or VI (α1, α2 and α3 chains) collagen [3, 43] or defective glycosaminoglycan formation [44, 45].

##### Skeletal radiographs in adults

To our knowledge, 33 cases of HFS have been reported in adults, of which 14 with X-ray findings. In 13 of these 14 cases, joint contractures, osteolytic destruction of the skull, of the large joints, of the long bones and of the extremities, triangular carpal bones and an isolated cortical erosion of mandibular bone and calcifications in the subcutaneous tumors were noted [4, 8, 16, 22, 25, 42, 46–52]. In one patient, no calcifications or bone involvement were noted on radiography [53].

Magnetic resonance imaging (MRI) of HFS lesions has rarely been described in adults and shows a hypointense, central, radiating scar and heterogeneous signal intensity. After the administration of a gadolinium contrast medium, the lesion showed diffuse enhancement, with the exception of the central scar and discrete enhancement of subcutaneous masses in contrasted phases [8, 51, 53].

Computed tomography (CT) of the head has demonstrated a normal aspect [51] or an abnormal bucco-lingual expansion with lingual cortical erosion [46], calcifications within the subcutaneous tumors, and a soft tissue mass extending from the hard palate into the nasal cavity and maxillary sinus [42]. Enhanced CT has revealed dye uptake in the submandibular and cervical lymph nodes bilaterally [42]. Brain CT has shown small ischemic regions in the right periventricular aspect, mild brain atrophy and extracranial tumor masses in the soft tissues of the right peritemporal and occipital aspects [47].

##### Histopathology

The histopathologic features of this disease include a normal epidermis with few inflammatory cells in the dermis and minor pigmentary incontinence. Deposits of an amorphous, homogeneous and eosinophilic, hyaline substance (periodic acid–Schiff positive), can be found in the papillary and reticular dermis, accompanied by a proliferation of spindle cells without atypia [10, 22].

Electron microscopic studies have shown stromal deposits of a fibrillogranular material focally displaying a banding pattern similar to that of type VI collagen and fibroblasts with prominent Golgi complexes, dilated endoplasmic reticulum, multi vesicular bodies and vesicles filled with a fibrillogranular material [3, 10, 43]. Calcospherules, defined as calcium-containing lamellar body have been described in JHF by Ko and Barr in 2003 [54].

##### Intestinal biopsy and imaging

The results of biopsy cases from patients with gastrointestinal signs include villous atrophy, edema, lymphangiectasia and hyalinosis. Rapid transit time has been described in real-time upper-gastrointestinal imaging investigations [55].

##### Immune system deficit

Deficits of the humoral and cellular branches of the immune system have been observed [56].

##### Laboratory studies

Laboratory examination may demonstrate a normal [22, 42, 57, 58] or abnormal aspect, such as an elevation of the Erythrocyte Sedimentation Rate (ESR) [47, 51, 59], thrombocytosis [60], mild anemia [4, 47, 51], or an elevation of serum albumin [61] or alkaline phosphatase [62].

#### Molecular diagnosis

*ANTXR2* is the only gene in which pathogenic variants are known to cause HFS. Mutations of this gene disrupt the formation of basement membranes. This disruption may allow the hyaline material to leak from plasma components through the basement membrane into the perivascular space, thus explaining the histological features of HFS [15].

As shown in Table 3, 41 different *ANTXR2* mutations have been described. Yan et al. [63] have reported that three frameshift mutations (c.1073-1074insC, c.1073-1074insCC and c.1074delT) represent approximately 60% of all pathogenic alleles. The incidence of insertions and deletions at positions 1073–1074 is probably due to its proximity to a low-complexity, GC-rich region encoding a stretch of proline residues that may constitute a vulnerable site for errors during DNA replication. The mutation p. Gly116Val identified in all patients in this study has previously been reported by Tümer et al. [64] in an 11-month-old Turkish girl with HFS. This mutation is located in the vWA domain and may damage ligand binding, not plasma membrane targeting, thus causing a severe manifestation of HFS. A comparison between the clinical signs of the patients in this study and the girl with the same mutation shows some differences: the girl presented with short stature and gingival hypertrophy and developed recurrent infections. She did not present with any visceral involvement. X-ray images did not show any osteolytic lesions [64]. These differences may be explained by the differences in age, and/or environmental factors.

**Table 3:** HFS mutations reported in the literature (updated from Denadai et al. and Deuquet et al.) [19, 22]

Mutation	Location	Domain	Protein	Hom/Het	Phenotype/grading system^a^	Ethnicity	Reference
1) c.2 T > G	Exon 1	Signal peptide	p.M1R	Hom	ISH/3	Dominican Republican	Antaya et al. [75]
2) c.116G > T	Exon 1	vWFA	p.C39F	Het	ISH/2 (patient1)	N.D.	Deuquet et al. [18]
3) c.134 T > C	Exon 1	vWFA	p.L45P	Hom	ISH/3 (family R)	Bedouin	Hanks et al. [15]
				Hom	ISH/4	Saudi	Mohamed et al. [61]
4) c.148G > A	Exon 1	vWFA	p.D50N	Hom	ISH/?	N.D.	Deuquet et al. [19]
5) c.225- 4G > A	Intron 2	vWFA	Presumed splice defect	Hom	ISH/3	Indian	Fong et al. [76]
6) c.277_278insATTATTT	Exon3	vWFA	p.L93Yfs^a^14	Hom	ISH/3	Indian	Koonuru and Venugopal [77]
						Indian	Aggarwal et al. [78]
7) c.304_305insA	Exon 4	vWFA	p.I102Nfs^a^12	Het	ISH/3	Chinese	Huang et al. [79]
8) c.314G > A	Exon 4	vWFA	p.G105D	Hom	JHF/3 (family JHF1)	Turkish	Dowling et al. [14]
9) c.347G > T	Exon 4	vWFA	p.G116V	Hom	ISH/3	Turkish	Tümer et al. [64]
				Hom	JHF/2 (patients VI-4, VI-5, VI-10 et V-15) ISH/3 patient VI-3	Lebanese	This report
10) c.353C > A	Exon 4	vWFA	p.T118K	Hom	ISH/4	Mexican	Lindvall et al. [80]
11) c.487-2A > G	Intron 5	vWFA	p.A163_Q185del; p.A163_K164del	Het	ISH/3 (Patient 5)	Brazilian	Denadai et al. [22]
12) c.495_496insA	Exon6	vWFA	p.S166Ifs^a^7	Hom	ISH/3 (family N and O)	Moroccan and Pakistani	Hanks et al. [15]
13) c.566 T > C	Exon 7	vWFA	p.I189T	Het	ISH/2 (family ISH2)	Swiss	Dowling et al. [14]
				Het	ISH/3 (family L)	European/Swiss	Hanks et al. [15]
14) c.652 T > C	Exon 8	vWFA	p.C218R	Hom	ISH/3 (family K)	Fijian/East Indian	Hanks et al. [15]
15) c.658G > T	Exon 8	vWFA	p.E220X	Hom	ISH/4 (family ISH1)	Turkish	Dowling et al. [14]
16) c.697 + 1dupG	Intron 8	Ig-like domain	G232insG	Het	ISH/?	N.D.	Deuquet et al. [19]
17) c.697 + 1 G > A	Intron 8	Ig-like domain	Presumed splice defect	Het	ISH/3 (family I)	European/Canadian	Hanks et al. [15]
18) c.796 + 2 T > C	Intron 9	Ig-like domain	Presumed splice defect	Het	JHF/2 (family E)^¥^	European	Hanks et al. [15]
19) c.867_945del	Exon 11	Ig-like domain	p.E289Dfs^a^22	Hom	ISH/4	Omani	Al Sinani et al. [81]
20) c.876_877insCAA	Exon 11	Ig-like domain	p.D292_VinsQ	Hom	JHF/2 (family G)	East Turkish	Hanks et al. [15]
21) c.928G > T	Exon 11	Ig-like domain	p.V310F	Het	ISH/2 (patient2)	N.D.	Deuquet et al. [18]
22) c.945 T > G	Exon 11	Ig-like domain	p.C315W	Hom	ISH/4 (patient 3)	N.D.	Deuquet et al. [18]
c.946-2A > G	Intron11	Ig-like domain	Presumed splice defect	Hom	ISH/4 (patient 1)	Iranian	Youssefian et al. [82]
23) N.D.	Exon11/12	Ig-like domain- Transmembrane	Presumed deletion	Hom	ISH/?	N.D.	Deuquet et al. [19]
24) c.986 T > G	Exon 12	Transmembrane	p.L329R	Hom	JHF/1 (family JHF2)	African American	Dowling et al. [14]
25) c.1073_1074insC	Exon 13	Cytoplasmic	p.A359Cfs^a^13	Het	ISH/2 (family ISH2)	Swiss	Dowling et al. [14]
26) c.1073_1074insC	Exon 13	Cytoplasmic	p.A359Cfs^a^13	Het	ISH/3	N.D.	Rahvar et al. [83]
				Het	ISH/3 (family J)^¥^	Chinese	Hanks et al. [15]
				Het	ISH/3 (family M)^¥^	Puerto Rican + African American	Hanks et al. [15]
				Hom	ISH/3 (family P)	United States, Hispanic	Hanks et al. [15]
				Hom	ISH/3	Taiwanese	Lee et al. [60]
				Hom	ISH/4 (patient 1)	Mexican	Shieh et al. [84]
				Het	ISH/3 (patient 3)		
				Het	ISH/4 (patient 4)	Brazilian	Denadai et al.[22]
				Het	ISH/2 (patient 2)	N.D.	Deuquet et al. [18]
				Hom	ISH/3 (patient4)	N.D.	Deuquet et al. [18]
				Het	ISH/3	Japanese	Sugiura et al. [85]
				Hom	ISH/2	North Indian	Narayanan and Phadke, [58]
				Hom	ISH/3 (patient 2)	Iranian	Youssefian et al. [82]
27) c.1073_1074insCC	Exon 13	Cytoplasmic	p.A359Lfs^a^51	Het	ISH/3 (family L)	European/Swiss	Hanks et al. [15]
28) c.1074delT	Exon 13	Cytoplasmic	p.A359Hfs^a^50	Hom	ISH/3 (family Q)	Kuwaiti	Hanks et al. [15]
				Hom	ISH/3	Iranian	Vahidnezhad et al. [86]
				Het	ISH/3	Chinese	Huang et al. [79]
				Het	ISH/3	Japanese	Hatamochi et al. [39]
				Hom	ISH/4 (family 1)	Egyptian	El-Kamah et al. [87]
				Hom	ISH/2	Moroccan	Jaouad et al. [9]
				Het	ISH/3 (patient 2 and 5)	Brazilian	Denadai et al. [22]
				Het	ISH/2 (patient3)	Brazilian	Denadai et al. [22]
				Het	ISH/4 (patient4)	Brazilian	Denadai et al. [22]
				Het	ISH/2 (patient1)	N.D.	Deuquet et al. [18]
				Hom	ISH/4 (family1)	Egyptian	El Kamah et al. [87]
				Hom	ISH/2 (patient3)	Iranian	Youssefian et al. [82]
29) c.1075insT	Exon 13	Cytoplasmic	p.A359Vfs^a^13	Het	ISH/?	N.D.	Deuquet et al. [19]
30) c.1086 + 1G > A	Intron13	Cytoplasmic	p.V394Ifs^a^6	Hom	JHF/2 (families C and F)	Turkish/European	Hanks et al. [15]
31) c.1087_1706del^∞^	Intron 13-17	Cytoplasmic	Presumed deletion	Hom	ISH/4 (patient 2)	Mexican	Shieh et al. [84]
32) c.1142A > G	Exon 14	Cytoplasmic	p.Y381C	Hom	JHF/1 (family D)	Moroccan	Hanks et al. [15]
				Hom	ISH/3	Moroccan	Mallet et al. [88]
33) c.1150C > T	Exon 14	Cytoplasmic	p.R384X	Het	ISH/3 (family I)	European/Canadian	Hanks et al. [15]
34) c.1156G > T	Exon 14	Cytoplasmic	p.V386F	Hom	JHF/2	Turkish	Hakki et al. [72]
35) c.1179G > A	Exon 14	Cytoplasmic	p.E363_E393del	Hom	JHF/2 (families A and B)	Indian	Hanks et al. [15]
36) c.1179 + 1 G > A	Intron 14	Cytoplasmic	Presumed splice defect	Hom	ISH/3	Chinese	Wang et al. [89]
37) c.1179 + 5G > T	Intron 14	Cytoplasmic	Presumed splice defect	Hom	JHF/2 (family H)	Turkish	Hanks et al. [15]
38) c.1180_1428del	Introns 14–16	Cytoplasmic	p.V394_E476del	Het	ISH/3 (patient 2) ISH/2 (patient3)	Brazilian	Denadai et al. [22]
39) c.1181 T > C	Exon 14	Cytoplasmic	p.V394A	Het	ISH/3	Japanese	Hatamochi et al. [39]
40) c.1294C > T	Exon 15	Cytoplasmic	p.R432X	Het	ISH/3 (patient 3)	Mexican	Shieh et al. [84]
				Het	ISH/3	N.D.	Rahvar et al. [83]
				Het	ISH/3	Japanese	Sugiura et al. [85]
41) c.1340delC	Exon 15	Cytoplasmic	p.P447Qfs^a^13	Hom	JHF/2 (family 2)	Egyptian	El-Kamah et al. [87]

*Hom* homozygous for mutation, *Het* heterozygous for mutation, *N.D.* not determinated

^a^Grading system according to classification of Nofal et al., and Denadai et al., [17, 22]

^¥^Compound heterozygote with only 1 mutation found

^∞^This is presumed since patient DNA could not be amplified for this region of the gene

### Mode of inheritance and genetic counseling

An autosomal recessive mode of inheritance has been established for HFS. Therefore, the risk for a parental carrier to have an affected offspring is 25%.

### Treatment and follow-up

Currently, only symptomatic treatments for HFS are available. Early surgical excision of the lesions is recommended for functional and cosmetic improvement [9, 52]. However, the lesions may recur and new lesions may appear [4, 52, 65, 66]. Intralesional steroid injections have been suggested because they can reduce the size of early lesions [9]. Capsulotomy, physiotherapy, treatment with cortisone and adrenocorticotropin (ACTH) have found modest success in the treatment of joint contractures [67]. Radiotherapy is not effective [10, 68]. Oral D-penicillamine may improve joint mobility and flexibility [65, 69]. Nonsteroidal anti-inflammatory drugs and opiates may be used to control pain and improve the quality of life [9, 70]. Gingival hyperplasia requires special dental care and many dental consultations to promote strict oral hygiene [71]. Gingivectomy may improve the quality of oral hygiene and nourishment by improving mastication and preventing gingival blood loss [72, 73]. Ablative laser surgery may be a reasonable choice and may be useful, at least as an adjunctive treatment [74]. In context of genetic therapies for this debilitating disorder, Deuquet et al. [18] have revealed that proteasome inhibitors may be potential therapeutic drugs for HFS patients with mutations in the ectodomain of *ANTXR2*.

## Conclusions

We report a Lebanese family with five adult patients with HFS. WGS showed an ability to establish a diagnosis in such puzzling cases in which the clinical signs are atypical or very severe for the classical phenotype. HFS is still a poorly understood disease with a severity that ranges from being lethal during early childhood to chronic at a later age. More studies to find an effective treatment are essential. An accurate diagnosis of the disease requires an exhaustive analysis of the radiologic and histopathological clinical findings, and genetic studies are required for family planning and counseling.

## Abbreviations

ACTH: Adrenocorticotropin
*ANTXR2*: Anthrax toxin receptor 2 gene
*CMG2*: Capillary morphogenesis protein gene-2
CT: Computed tomography
ESR: Erythrocyte Sedimentation Rate
ExAC: Exome Aggregation Consortium
HFS: Hyaline fibromatosis syndrome
Ig-like: Immunoglobulin–like domain
ISH: Infantile systemic hyalinosis
JHF: Juvenile hyaline fibromatosis
MRI: Magnetic resonance imaging
NGS: Next-Generation-Sequencing
TM: Transmembrane
vWA: von Willebrand A
WGS: Whole Genome Sequencing
*ZNF618*: Zinc finger protein 618
