# Erratum to: Diagnosis implications of the whole genome sequencing in a large Lebanese family with hyaline fibromatosis syndrome

**DOI:** 10.1186/s12863-017-0480-z

**Published:** 2017-02-01

**Authors:** Zahraa Haidar, Ramzi Temanni, Eliane Chouery, Puthen Jithesh, Wei Liu, Rashid Al-Ali, Ena Wang, Francesco M Marincola, Nadine Jalkh, Soha Haddad, Wassim Haidar, Lotfi Chouchane, André Mégarbané

**Affiliations:** 1 Unité de Génétique Médicale, Faculté de Médecine, Université Saint-Joseph, Beirut, Lebanon; 2 Bioinformatics Division, Sidra Medical & Research Center, Doha, Qatar; 3 Genomics Core Laboratory, Translational Medicine Division, Sidra Medical & Research Center, Doha, Qatar; 4 Research office, Sidra Medical & Research Center, Doha, Qatar; 5 Department of Radiology, Hotel Dieu de France University hospital–Beirut, Beirut, Lebanon; 6 Department of General surgery, Dar Al Amal University Hospital-Baalbeck, Baalbeck, Lebanon; 7 Laboratory of Genetic Medicine and Immunology, Weill Cornell Medicine-Qatar, Doha, Qatar; 8 Institut Jérôme Lejeune, 37, rue des Volontaires, Paris, 75015 France

## Erratum

Shortly after the publication of this article [1], one of the authors noticed that his name had been misspelled. The article has been updated. ‘Puthen Jitesh’ has been changed to ‘Puthen Jithesh’.
